# Gender Inequalities in Employment of Parents Caring for Children With Autism Spectrum Disorder in China: Cross-Sectional Study

**DOI:** 10.2196/59696

**Published:** 2024-12-17

**Authors:** Yanan Zhao, Huiyun Fan, Yanan Luo, Rong Zhang, Xiaoying Zheng

**Affiliations:** 1 School of Public Health, Peking University, Beijing, China; 2 Faculty of Health and Wellness, City University of Macau, Macau, China; 3 School of Population Medicine and Public Health, Chinese Academy of Medical Sciences, Peking Union Medical College, Number 31, Road 3rd, Bei-Ji-Ge, Dongcheng District, Beijing, 100730, China, 86 13621224975; 4 Department of Global Health, School of Public Health, Peking University, Beijing, China; 5 Neuroscience Research Institute, Peking University, Beijing, China; 6 Department of Neurobiology, School of Basic Medical Sciences, Peking University Health Science Center, Beijing, China; 7 Key Laboratory for Neuroscience, Ministry of Education and National Health Commission, Beijing, China; 8 Autism Research Centre, Peking University Health Science Centre, Beijing, China

**Keywords:** autism spectrum disorder, family, employment status, influencing factors, autism, child care, children, China, parent, online survey, mother, father, adolescent, youth, ASD, children with autism

## Abstract

**Background:**

The increasing need for child care is placing a burden on parents, including those with children with autism.

**Objective:**

The aim of this study was to examine the employment status of Chinese mothers and fathers with children with autism spectrum disorder (ASD), as well as to investigate the factors that affected their employment decisions.

**Methods:**

An online national survey was completed by the parents of 5018 children and adolescents with ASD aged 2-17 years (4837 couples, 181 single mothers, and 148 single fathers). The dependent variable was employment status—whether they kept working or quit to take care of their child. The independent variables were those characterizing the needs of the child and the sociodemographic characteristics of the family.

**Results:**

The employment rate of mothers with children and adolescents with ASD was 37.3% (1874/5018), while 96.7% (4823/4988) of fathers were employed. In addition, 54.3% (2723/5018) of mothers resigned from employment outside the home to care for their children, while only 2.8% (139/4988) of fathers resigned due to caring obligations. Mothers’ employment was positively associated with their single marital status, lower educational level, and having assistance from grandparents. Having the grandparents’ assistance was positively associated with fathers’ employment.

**Conclusions:**

Gender inequalities in employment exist in China. Mothers caring for children with ASD had lower workforce participation than fathers. More female-friendly policies and a stronger gender equality ideology would be of benefit to Chinese society.

## Introduction

Autism spectrum disorder (ASD) encompasses a range of neurodevelopmental disorders that are characterized by the following core deficits: (1) impairments in social interaction and communication, and (2) restricted, repetitive behaviors [1]. Due to the child’s significant needs, daycare availability may be limited, leading to a parent needing to stay at home [2]. Comparative research suggests that parents of children with ASD spend more time providing care for their children at home than those of children without special health care needs [3]. There is strong evidence suggesting that having a child with ASD in the household adversely affects the employment status of the parents [4,5]. In the United States, approximately 30%-50% of caregivers of children with ASD have completely ceased working [6]. Studies have shown that the employment of parents of children with ASD is disproportionately affected when compared to families with children who have other disabilities [7,8]. In 2-parent households, the employment of mothers is disproportionately affected [9,10]. In Europe and the United States, mothers, compared to their partners, are more likely to hold part-time, lower-status positions that do not offer commensurate compensation corresponding to their educational qualifications [11-13]. A systematic review and a case-control study in Israel showed that working mothers of children with ASD were at increased risk of not maintaining their working status over the 5 years following their child’s birth [14,15]. It reported that US mothers of children with ASD were less likely to be employed and earned 56% less than mothers of children without ASD; simultaneously, there was no significant impact on the fathers’ labor market participation [4].

Globally, women’s labor participation and outcomes still fall behind those of men, primarily due to persistent gender inequality resulting from the impact of childbirth [16], which can be seen as a “motherhood penalty” [17]. It can doubly penalize mothers of children with ASD, as they face challenges related to both gender and their child’s condition. Variations in family policies and societal gender norms across countries contribute to diverse circumstances for mothers. Scandinavian countries such as Denmark and Sweden demonstrate the lowest levels of the “motherhood penalty,” while the United Kingdom and the United States fall in the middle, and Germany and Australia have the highest penalties [18].

Cultural factors, such as the traditional concept of “men outside and women inside” commonly found in East Asian cultures [19,20], as well as the evolving social norms influenced by market economics that emphasize the contributions of both women and men in Chinese society [21], may contribute to variations in the Chinese context. From a societal standpoint, higher rates of female labor force participation within extended families were observable in China [22,23], which was less prevalent in Europe and the Americas [24]. In China, there are no guaranteed free services; the significant economic burden this represents may drive women to seek employment. However, inadequate educational and childcare resources, along with inflexible employment arrangements, may compel women to leave the workforce after having a child with ASD. Unfortunately, the limited available evidence from China prevents a thorough examination of this topic. The employment situation for families with children with ASD remains uncertain, particularly in terms of gender dynamics, and the underlying mechanisms are not fully understood. Gender disparities in employment can be elucidated through 2 theories. First, the specialization theory suggests that the division of paid and unpaid work is a rational contract between partners driven by utility maximization [25]. The partner with the lower salary is expected to do more housework. Second, the gender role perspective posits that the ability to balance work and parenthood is influenced by individuals’ identities as moral beings within their culture [26]. Different cultures may vary in their approach to these issues. Gaining a better understanding of the situation in China is crucial, not only for informing policymaking within the country, but also for providing valuable insights into the cultural ramifications. There are many factors that affect whether caregivers work or not. Previous research has found that caregivers’ labor force participation is influenced by both individual factors (eg, child age, caregivers’ age, educational level, marital status, the severity of ASD symptoms, or caregivers’ medical conditions) and the availability of childcare resources or perceived social support [4,27-29]. Compared to mothers of children without exceptional health care needs, US mothers of children with mild ASD and moderate/severe ASD had a 12% and 25% lower probability of being employed, respectively [30]. Although literature from other countries has provided valuable insights, current research in this area still has limitations. First, there is a lack of in-depth discussion on gender differences in employment. Many studies focus on the impact of caregiving from a female perspective, ignoring gender inequalities within families. As most of the caregivers in these studies were mothers of children with ASD, the results may not be generalizable to fathers [31]. Second, there is a need for a better understanding of the factors associated with the employment of both mothers and fathers. By examining the factors that influence employment, policymakers can better target interventions toward the most vulnerable populations. Third, most studies have had small sample sizes and have predominantly focused on very young children. A broader age span sample can help capture the situation of families at different stages.

Parental employment in families with children with ASD is an area of particular significance. The employment status of parents in these families plays a vital role in reducing family stress, accessing essential resources, and promoting the well-being of both parents and children [32-34]. In this study, the primary goal was to uncover the employment status of parents in families with children and adolescents with ASD. The second goal was to investigate the factors associated with employment of mothers and fathers caring for children with ASD in China. The following assumptions were made: (1) parents’ employment decisions can be influenced by both parental and child traits, and (2) there are differences between the factors influencing fathers’ and mothers’ employment decisions. A comprehensive understanding of how these factors influence parental employment could inform the development of effective policies to support these families.

## Methods

### Participants

This study used secondary data from the Survey on Family Circumstances and Demand for Support and Resources among Autistic Children in China in 2020. It was a survey that was distributed to members of an online parent community of children with ASD or other developmental disorders in China. The questionnaire was designed to collect information on various aspects of the families. Parents were asked to provide information about their employment status, including current work status, work history, and any work adjustments made due to their child’s needs. Additionally, parents reported on their child’s diagnosis. The other details of the survey have been described elsewhere [5]. A pilot field study (N=20) was conducted to refine the instrument and data collection procedures, and the results indicated that respondents generally understood the questionnaire, so only minor wording changes were made.

### Data Collection

Families with children diagnosed with ASD were recruited if they met the following criteria: (1) they were between the ages of 2 and 17 years and were diagnosed with or suspected (children under 3 years old cannot be officially diagnosed) of having ASD at a hospital, and (2) the hospital had diagnostic qualifications and followed a Diagnostic and Statistical Manual of Mental Disorders, Fifth Edition standard, not only through scale measurement but also via diagnosis by a medical professional. Exclusion criteria were individuals with ASD with severe comorbidities such as physical disability and cerebral palsy. The samples with obvious errors or omissions were also excluded in this study. Regarding omissions, we used the following strategies: (1) we needed complete data for the major variables that were essential to our primary analysis, and (2) we permitted some missing data for secondary variables and used multiple imputation to account for these omissions. Any questionnaire with a missing data rate exceeding 33% was excluded from the analysis. For questionnaires with a lower missing data rate, we used multiple imputation to handle missing values in noncore variables, maximizing the retention of data information. There were 8014 households investigated, with 5018 (62.62%) households included. This survey’s gender ratio and family location distribution were consistent with China’s overall population distribution. In total, 31 Chinese provinces and 380 cities or districts were included (see Table S1 and Table S2 in the Multimedia Appendix 1 for details).

### Measures: Assessments of Employment Status

When assessing workforce participation, we used the primary categories of “work” and “nonwork” (as a stay-at-home parent). We used the term “nonwork” in this study to refer to parents who were either unemployed or out of the labor force. In order to further distinguish the effects of different employment types, this study constructed 4 employment status variables, namely “full-time,” “flexible,” “overtime,” and “a long leave of absence.” Full-time work generally refers to a person being formally employed, working a minimum of 40 hours per week, and enjoying corresponding wages, social insurance, and welfare benefits (as described in the Labor Law of the People’s Republic of China). Flexible work refers to employment for which working hours (usually less than 30 hours per week) or working places are not fixed. Overtime work refers to working more than 40 hours per week. We created 2 variables for full-time parents so as to understand the reason for nonwork: “caregiving resignation” and “other.” Caregiving resignation meant that the parent resigned after their child was diagnosed with ASD and that they were taking care of the child. Resigning due to caregiving responsibilities, which is considered an “involuntary resignation” or “involuntary unemployment,” was considered as being beyond one’s control. “Other” refers to those who resigned before their child’s diagnosis of ASD or those who remained unemployed for other reasons after the child’s birth (see Table S3 in the Multimedia Appendix 1 for definitions). In other words, “not working” referred to the current employment status, including those who resigned due to childcare needs and those who were not working for other reasons (including those who never worked). “Resigning” specifically referred to leaving a job due to childcare needs for their child with ASD.

### Socioeconomic and Demographic Variables

The age of the children was their age at the survey point. The age of the children was divided into four age groups: 2‐5 years, 6‐9 years, 10‐13 years, and 14‐17 years. The severity of ASD was judged according to professional evaluation or the parents’ subjective judgment. There were four categories: low-functioning autism (LFA), middle-functioning autism, high-functioning autism (HFA), and undetermined. The regional variables were “eastern,” “central,” and “western.” The provinces in the eastern region were among the first to implement the coastal opening-up policy and have a high level of economic development. The provinces of the central region are economically underdeveloped, while those of the western region are even less so. We classified family income into 3 categories. According to the data distribution, the below-average group had an annual income of less than US $12,327 (80,000 yuan), the around-average group had an annual income of between US $12,327 (80,001 yuan) and US $23,112 (150,000 yuan), and the above-average group had an annual income of more than US $23,112 (150,000 yuan). Whether the children’s grandparents were able to provide assistance was one way to measure informal social supports, so “grandparents’ assistance” was included. The term “physical health status” was used to describe negative physical health status. We asked respondents about their subjective physical status using a 5-point Likert scale with 1=“Extremely poor,” 2=“Poor,” 3=“Average,” 4=“Good,” and 5=“Very good” in response to the following question: “How do you evaluate your physical health generally?” The question referred to the 4 weeks preceding the survey. The answers to the items were divided, with 2 being the cutoff score for poor physical health status. A single-item question was used in previous research [35]; this single item has been reported to be reliable. Other background information was collected on children’s gender, whether they have comorbidities or not, and parents’ education and marital status.

### Statistical Analysis

The mean (SD) of normally distributed data was used. The comparisons of characteristics (categorical) were analyzed using *χ^2^* tests and the comparisons of characteristics (continuous) were analyzed using *F* tests. Logistic regression models were used to identify the factors influencing employment status. Associations between predictors and independent variables were reported using odds ratios (ORs) and their 95% CIs. Group comparisons were made between mothers and fathers. All the estimated costs were converted to US dollar (US $) values in January 2021, when US $1 was equivalent to about 6.49 Chinese yuan. All statistical analyses were conducted using SPSS for Windows (version 22.0; IBM Corp).

### Ethical Considerations

All families provided electronic informed consent before enrollment. All procedures involving human subjects/patients were approved by the ethics committee of the Peking University Institutional Review Board (approval number IRB00001052-20016). The informed consent process clearly outlined the study's purpose, procedures, and potential risks, with participants retaining the right to opt out at any time. Participant data were anonymized using unique identifiers to protect individual privacy. No direct financial compensation was provided, and no identifiable features of research participants were included in any research materials.

## Results

### Sample Descriptive Statistics

**Figure 1.**
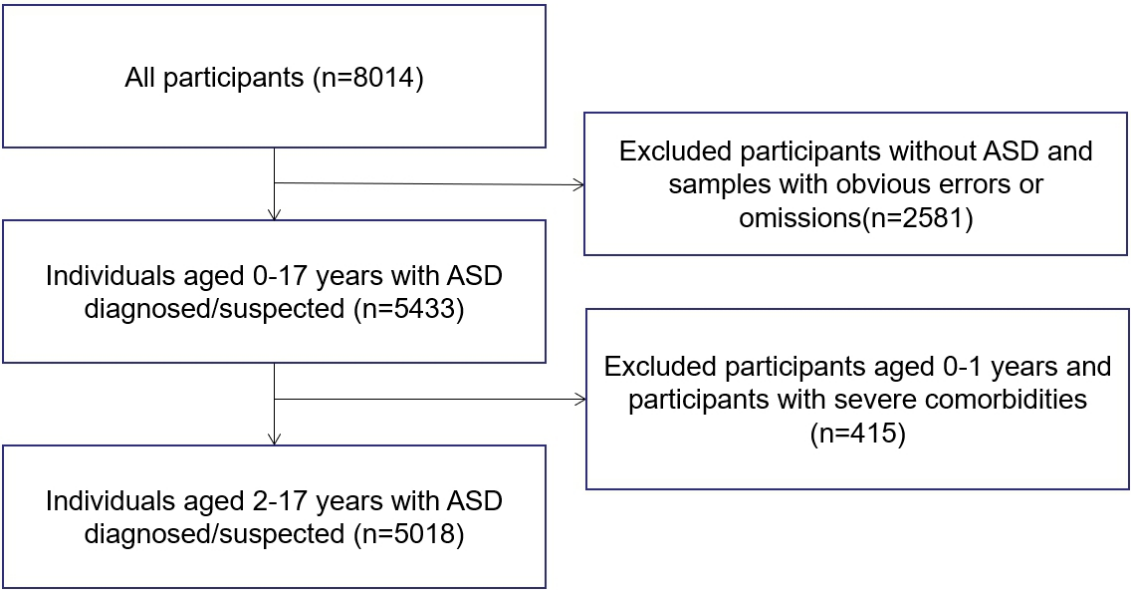
Flowchart of the selection procedure. ASD: autism spectrum disorder.

In total, 5018 households were included in this survey (Figure 1). Most of the children (4227/5018, 84.2%) were boys, and the mean age was 5.3 (SD 2.6) years old, with the leading ASD severity being middle function (2030/5018, 40.5%). In addition, 18.2% (913/5018) of the children had comorbidities such as attention-deficit/hyperactivity disorder and epilepsy. Most of the parents had a college degree (3430/5018, 68.4%) and 31.1% (1563/5018) of the parents received assistance from the children’s grandparents. Mothers had a lower rate of employment than fathers. This survey revealed disparities regarding whether parents were employed, classified by the severity of ASD, parents’ education level, whether parents were single, whether grandparents could give assistance, family income, and other factors. The results of the group comparisons can be seen in Table 1. There were statistically significant differences between the working and nonworking groups for parents (*P*<.05). From the perspective of whether children had comorbidities, there was no statistically significant difference between the parent working and nonworking groups (*P*<.05). The study population is further described in Table 1.

**Table 1.** Characteristics of children, fathers, and mothers.

Characteristics	Overall, n (%)	Mothers (n=5018), n (%)		Chi-square (*df*)	*P* value	Fathers (n=4988), n (%)		Chi-square (*df*)	*P* value
		Employed (n=1874)	Unemployed (n=3144)			Employed (n=4823)	Unemployed (n=165)		
**Children’s characteristics**									
***Age groups (years)***				16.351 (3)	.001			9.299 (3)	.12
2‐5	3119 (62.2)	1156 (61.7)	1963 (62.4)			3003 (62.3)	98 (59.4)		
6‐9	1527 (30.4)	545 (29.1)	982 (31.2)			1472 (30.5)	47 (28.5)		
10‐13	309 (6.2)	140 (7.5)	169 (5.4)			287 (6)	19 (11.5)		
14‐17	63 (1.3)	33 (1.8)	30 (1)			61 (1.3)	1 (0.6)		
***Gender of child***				3.513 (1)	.03			1.824 (1)	.10
Male	4227 (84.2)	1602 (85.5)	2625 (83.5)			4056 (84.1)	144 (87.3)		
Female	791 (15.8)	272 (14.5)	519 (16.5)			767 (15.9)	21 (12.7)		
***Autism spectrum disorder severity***				115.284 (3)	<.001			1.805 (3)	.33
Low-functioning autism	1140 (22.7)	337 (18)	803 (25.5)			1088 (22.6)	44 (26.7)		
Middle-functioning autism	2030 (40.5)	730 (39)	1300 (41.3)			1955 (40.5)	64 (38.8)		
High-functioning autism	945 (18.8)	488 (26)	457 (14.5)			910 (18.9)	30 (18.2)		
Undetermined	903 (18)	319 (17)	584 (18.6)			870 (18)	27 (16.3)		
***Comorbidity***				0.363 (1)	.97			1.093 (1)	.17
Yes	913 (18.2)	333 (17.8)	580 (18.4)			872 (18.1)	35 (21.2)		
No	4105 (81.8)	1541 (82.2)	2564 (81.6)			3951 (81.9)	130 (78.8)		
**Parents’ characteristics**									
***Education***				781.216 (1)	<.001			0.690 (1)	.22
College degree or above	3430 (68.4)	1657 (88.4)	1773 (56.4)			3302 (68.5)	108 (65.5)		
High school or below	1588 (31.6)	217 (11.6)	1371 (43.6)			1521 (31.5)	57 (34.5)		
***Marital status: single parent***				11.223 (1)	.001			9.739 (1)	.004
Yes	181 (3.6)	89 (4.7)	92 (2.9)			166 (3.4)	13 (7.9)		
No	4837 (96.4)	1785 (95.3)	3052 (97.1)			4657 (96.6)	152 (92.1)		
***Grandparents’ assistance***				870.885 (1)	<.001			19.142 (1)	<.001
Yes	1563 (31.1)	1052 (56.1)	511 (16.3)			1530 (31.7)	28 (17)		
No	3455 (68.9)	822 (43.9)	2633 (83.7)			3293 (68.3)	137 (83)		
***Self-reported physical state: poor***				9.928 (1)	.002			6.176 (1)	.01
Yes	788 (15.7)	255 (13.6)	533 (17)			745 (15.4)	36 (21.8)		
No	4230 (84.3)	1619 (86.4)	2611 (83)			4078 (84.6)	129 (78.2)		
***Household income***				289.321 (2)	<.001			29.869 (2)	<.001
Below average	1556 (31)	332 (17.7)	1224 (38.9)			1252 (26)	86 (52.1)		
Around average	1627 (32.4)	576 (30.7)	1051 (33.4)			1696 (35.2)	46 (27.9)		
Above average	1835 (36.6)	966 (51.5)	869 (27.6)			1875 (38.9)	33 (20)		
***Resident district***				30.908 (2)	<.001			1.828 (2)	.40
Eastern	3061 (61)	1163 (62.1)	1898 (60.4)			2950 (61.1)	94 (57)		
Central	1483 (29.6)	489 (26.1)	994 (31.6)			1417 (29.4)	56 (33.9)		
Western	474 (9.4)	222 (11.8)	252 (8)			456 (9.5)	15 (9.1)		

### Predictors of Employment for Fathers and Mothers

Mothers with children with HFA were more likely to be employed than mothers who had children with LFA (OR 1.94, 95% CI 1.46‐2.56; Table 2). Mothers who had a high school education or less were 3.64 times more likely to be employed than mothers who had a college degree or above (OR 3.64, 95% CI 2.89‐4.57). Single mothers had higher odds of having a job (OR 2.03, 95% CI 1.26‐3.08). Having assistance from children’s grandparents’ meant that both mothers and fathers were more likely to be employed (mothers: OR 5.57, 95% CI 4.67‐6.64; fathers: OR 2.31, 95% CI 1.39‐2.84). Children’s gender and age were not significantly associated with mothers’ and fathers’ employment status.

**Table 2.** Predictors of employment for fathers and mothers.

Charactistics	Mother employed, OR^a^ (95% CI)	Father employed, OR (95% CI)	Mother resigned, OR (95% CI)	Father resigned, OR (95% CI)
**Gender of child (reference: male)**				
Female	1.05 (0.84-1.32)	0.31 (0.77-2.22)	1.10 (0.90-1.35)	0.74 (0.41-1.34)
**Age of child, years (reference: 2-5 years)**				
6‐9	1.48 (0.83-2.63)	0.60 (0.23-1.54)	0.74 (0.44-1.24)	1.72 (0.60-4.89)
10‐13	1.01 (0.62-1.64)	0.61 (0.24-1.58)	0.99 (0.67-1.47)	1.14 (0.76-1.70)
14‐17	1.14 (0.76-1.71)	0.56 (0.22-1.41)	0.80 (0.48-1.33)	1.25 (0.62-2.55)
**Comorbidity (reference: no)**				
Yes	0.87 0.67-1.13)	0.77 (0.47-1.26)	1.06 (0.84-1.34)	1.15 (0.65-2.05)
**Autism spectrum disorder severity (reference: LFA^b^)**				
MFA^c^	1.18 (0.93-1.50)	1.04 (0.63-1.70)	0.93 (0.75-1.14)	0.93 (0.54-1.60)
HFA^d^	1.94 (1.46-2.56)	0.68 (0.38-1.21)	0.66 (0.51-0.86)	1.28 (0.67-2.45)
Undetermined	1.19 (0.91-1.55)	1.16 (0.66-2.03)	0.86 (0.68-1.08)	0.78 (0.47-1.47)
**Education (reference: college degree or above)**				
High school or below	3.64 (2.89-4.57)	0.68 (0.45-1.03)	0.58 (0.49-0.69)	1.50 (0.94-2.40)
**Marital status: single parent (reference: yes)**				
No	2.03 (1.26-3.08)	0.54 (0.25-1.16)	0.50 (0.32-0.80)	1.53 (0.59-3.53)
**Grandparents’ assistance (reference: no)**				
Yes	5.57 (4.67-6.64)	2.31 (1.39-2.84)	0.26 (0.21-0.30)	0.30 (0.16-0.56)
**Self-reported physical state: poor (reference: no)**				
Yes	0.96 (0.75-1.24)	1.06 (0.64-1.74)	1.35 (1.09-1.68)	1.08 (0.62-1.86)
**Household income (reference: below average)**				
Around average	1.50 (1.19-1.90)	2.65 (1.69-4.15)	0.79 (0.65-0.96)	0.46 (0.28-0.75)
Above average	2.04 (1.61-2.57)	4.00 (2.36-6.76)	0.52 (0.42-0.64)	0.35 (0.20-0.63)
**Resident district (reference: eastern)**				
Central	0.95 (0.78-1.15)	1.16 (0.77-1.74)	1.02 (0.87-1.22)	0.88 (0.55-1.38)
Western	1.51 (1.13-2.02)	1.35 (0.66-2.75)	0.70 (0.53-0.93)	0.87 (0.41-1.86)

a OR: odds ratio.

b LFA: low-functioning autism.

c MFA: middle-functioning autism.

d HFA: high-functioning autism.

### Resignations

We found that 37.3% (1874/5018) of children’s mothers were employed; of these, 12.4% (622/5018) had a flexible job, 4.6% (232/5018) took an extended leave, and 1% (49/5018) chose to increase working hours. However, the proportion of fathers working full-time was much higher (3537/4988, 70.9%), while 7.5% (372/4988) of fathers worked flexible hours and 14.9% (743/4988) of fathers worked overtime (Table 3). In addition, 3.4% (171/4988) of fathers took a long leave of absence. Only 3.3% (165/4988) of fathers were unemployed, with the majority citing child care as the main reason. We found that 2.8% (138/4988) of fathers and 54.3% (2723/5018) of mothers resigned because they needed to care for their child with ASD.

Mothers with children with HFA were less likely to resign to take care of their children than the mothers with children with LFA (OR 0.66, 95% CI 0.51‐0.86). Mothers who had no college degree were less likely to resign to take care of their children (OR 0.58, 95% CI 0.49‐0.69). Single mothers had lower odds of resigning to take care of their children (OR 0.50, 95% CI 0.32‐0.80). Mothers with poor physical health were more likely to resign to take care of their children (OR 1.35, 95% CI 1.09‐1.68). Having assistance from the children’s grandparents meant that both mothers and fathers were less likely to resign (mothers: OR 0.26, 95% CI 0.21‐0.30; fathers: OR 0.30, 95% CI 0.16‐0.56). Assistance from grandparents was the only factor influencing the fathers’ decision to resign in this model.

Taking “salary of the last job before resignation was higher than the average” and “previous income’s proportion of the total household income was higher than average” as independent variables, the logistic regression for involuntary resignation showed that even when mothers had a higher annual income from their previous employment or previously earned a higher proportion of the total household income, they were more likely to involuntarily resign. However, there was no such trend for fathers (Table 4). When we compared salaries between spouses, we found that 45.3% (63/139) of fathers who resigned to care for their children earned more than their spouses before resigning. On the other hand, only 13.7% (373/2723) of mothers who resigned to care for their children earned more than their spouses before resigning.

**Table 3.** Characteristics of employment status and reasons for not being employed by gender.

Characteristics	Father (n=5018), n (%)	Mother (n=5077), n (%)
**Work**		
Full-time	3537 (70.9)	971 (19.4)
Flexible	372 (7.5)	622 (12.4)
Overtime	743 (14.9)	49 (1)
Long leave	171 (3.4)	232 (4.6)
**Nonwork**		
Caregiving resignation	139 (2.8)	2723 (54.3)
Other	26 (0.5)	421 (8.4)

**Table 4.** The logistic regression for the higher income and proportion of household income for involuntarily unemployed parents.

Variables	Model 1 (n=2723, mothers), OR^a^ (95% CI)	Model 2 (n=139, fathers), OR (95% CI)	Model 3 (n=2723, mothers), OR (95% CI)	Model 4 (n=139, fathers), OR (95% CI)
Involuntary resignation	1.57^b^ (1.21‐2.03)	0.24 (0.12‐2.41)	1.25^c^ (1.01‐1.58)	0.55 (0.15‐6.83)
**Independent variable** ^**d**^				
Annual income from previous work was higher than average	√	√		
Previous annual income’s proportion of the total household income was higher than average			√	√

a OR: odds ratio.

b *P*<.001.

c *P*<.01.

d Models 1-4 controlled the age of the children, autism spectrum disorder severity, children having comorbidities or not, parents having college degree or not, parents’ marital status, and the family’s residence region and annual income. Independent variables were the salary of the last job before resignation being higher than the average (1=yes, 0=no) and its proportion being higher than the average (1=yes, 0=no).

## Discussion

### Principal Findings

This study provided a comprehensive analysis of the employment status and factors influencing employment among mothers and fathers who cared for children with ASD in China. It is the first study to investigate the gender differences in influencing factors among Chinese parents. The results of the study highlight a notable disparity, indicating that females in China are more likely to be unemployed and more likely to resign from their jobs due to child care responsibilities compared to males. This trend persists even as the children grow older and reach school age.

This research has shed light on the existence of gender inequalities in employment within the context of caring for children with ASD. That having a child has an impact on the mother’s job and career is not unusual, as evidenced by previous research [11,27,36-38]. Previous studies have reported that the employment rate for mothers of children with ASD was 67%, while for fathers, it was higher (92%) [14]. Additionally, it was found that 79% of fathers and 44% of mothers with children with ASD were engaged in full-time employment [39]. It is noteworthy that in China, the issue of gender inequality in employment may be particularly pronounced. In this study, it was found that only 37.3% of mothers of children with ASD were employed, while the employment rate for fathers was higher (96.7%). This finding was consistent with the trends found in previous studies [14,39], but the gender difference in employment rates was more pronounced. The employment rate for mothers in this study was even lower compared to previous findings from other countries for parents of children with developmental disabilities; for instance, the employment rates were reported as 49% in Japan [40]. The reasons could be summarized as a difference in cultures, diagnoses, and research designs. It should be noted that this study was conducted during the COVID-19 pandemic, a context that might have influenced our findings. The pandemic had a dual impact on employment opportunities for parents of children with ASD. First, the overall economic downturn might have reduced job prospects, particularly for those living in informal settlements who rely on informal, precarious employment with irregular income [41]. Second, increased caregiving demands, such as those resulting from school closures, might have further constrained these parents’ employment options [42].

Utilizing data from the China Family Panel Study spanning the years 2010 to 2018, research found that the employment rate for women was 68.5%, whereas for men, it was 93.3% [43]. Similarly, based on national survey data, the employment rate for Chinese women was 66.7%, with a lower rate of 51.4% observed for women with children aged 0‐3 years [44]. These findings highlight the disparities in employment rates between genders in China. The employment rate of women in our sample was lower than the average employment rate of women in China. Across nearly all age groups of children with ASD, the employment rate for mothers was below 40% in this study. The employment rate for fathers did not differ, suggesting that mothers were more likely to assume the primary caregiver role. These findings aligned with previous research that indicated that mothers were often the ones who exited the workforce when caring for a child with a disability [45,46].

For those mothers who were employed, they were less likely to work full-time or for longer working hours. Only 4.6% of mothers in this study took a long leave. In addition, while 15% of fathers reported working overtime, only 1% of mothers did. These findings highlighted the disparity in working hours and overtime between mothers and fathers. Fathers were still the primary breadwinners in their families. When a child has a disability, families must frequently make the difficult decision to have one parent leave their job to become the primary caretaker while the other parent continues to work. This decision is tough and challenging [47]. Two theories, namely the specialization theory proposed by Becker [25] and the gender role theory introduced by Finch [26], can help explain the gender inequalities observed in employment. Although our findings suggest that mothers’ lower relative salary compared to their spouses may play a role in their decision to take on primary caregiving responsibilities, it is likely that a combination of factors—including cultural norms, personal preferences, and family circumstances—also influence these decisions. There is evidence supporting both theories. How individuals balance work and parenthood is also influenced by individuals’ identities as moral beings within their specific culture. However, it is important to note that the applicability of this theory may vary across countries.

Some factors, including culture, household income, and women’s role identity, can influence women’s labor participation and types of participation, though the results are generally inconclusive [27]. In this study, we found that the severity of the ASD symptoms increased mothers’ caregiving needs and thus decreased employment, which was consistent with previous findings that the more severe the child’s condition, the more challenging it was for the parents to work [48,49]. For fathers, having children with more severe disabilities had little effect on their labor market participation, which was consistent with a previous finding [13]. There were similar findings in other studies [38,50]. The effect seems to be stronger among more educated mothers, signaling the existence of education inequalities in mothers, but not in fathers. Our study revealed that highly educated mothers were more prone to resign to care for their children. This was likely due to higher spousal incomes rather than a lack of social supports. In fact, these educated mothers were more likely to receive childcare assistance from the children’s grandparents. Our findings indicated that mothers with higher previous incomes were more likely to resign involuntarily, while those with lower incomes tended to continue working. This trend raises concerns for families with children with ASD, particularly regarding reduced access to intensive early interventions and the potential negative impact of work-related stress on parent-child interactions in dual-income households.

Social supports were an important influencing factor for employment for parents. The assistance provided by grandparents had a significant impact on the decision to work, especially for mothers. In traditional Chinese culture, parents of children with ASD may tend to seek help from their family rather than from other sources [19,51], but this does support mothers’ employment. In this study, we found that single mothers were more likely to work, supporting the previous finding that single mothers worked more out of economic necessity [52]. Single fathers, on the other hand, did not have this tendency to work more. One of the explanations for this was that fathers may face discrimination due to their single-parent status [53]. Another possible cause was Confucianism, which holds that men are superior to women. Fathers were more inclined to rely on their parents’ resources, which alleviated financial concerns [54]. Although we did not explicitly measure discrimination and parental resources in our study, it was plausible that this phenomenon existed among our participants. Additional research is necessary to substantiate these claims.

Previous research found that mothers who experience involuntary unemployment tended to have a lower quality of life [48]. It emphasized the critical role of mothers in caregiving as well as the potential negative impact of additional caregiving responsibilities. In the short term, to care for their children, women may have to interrupt or leave their careers, which can hinder their professional development. In the long term, women may experience a decline in their social status and decision-making power within the family. More research is still needed to verify this hypothesis in the future.

When it comes to addressing gender inequalities, there are valuable practices that can serve as examples. One such instance can be observed in Australia, where the motherhood penalty had been severe. However, the government implemented a range of proactive family policies to address this issue. There was a notable policy focus on supporting parents in their return to the workforce as soon as they were able. This was achieved through various measures, including investments in the improvement of childcare systems and the provision of increased subsidies for working parents [28]. Family policies that promote a dual earner-dual carer model have played a pivotal role in raising female employment rates and reducing gender disparities in employment [55]. These policies have proven to be particularly beneficial for women with lower and moderate levels of educational qualifications.

### Limitations

This study had a number of limitations. First, the sampling method was nonprobabilistic, relying on voluntary participation rather than random selection, which did not allow for control stratification in sampling. To improve future studies, we could use more robust sampling survey methodologies. Second, the design of the study prevents the establishment of causal relationships. We can only test correlations between influencing factors and parental employment. This research would benefit from more sophisticated modeling techniques to investigate the ways in which the variables interact. Third, the data regarding the severity of the ASD symptoms was not professionally approved using the validated scales but rather was based on parental reports. Improved symptom classification will facilitate the creation of more appropriate aids. Fourth, although parents of children with ASD share some commonalities with parents of children with other disabilities, it is not clear whether the current findings are applicable outside of this particular case. Further research is needed to understand the extent to which these differences are related to the structure and arrangement of services. Fifth, the data collection for this study was completed during the COVID-19 pandemic, which may have affected the generalizability of our findings. Although the results provide insights into the situation during this period, they may not fully represent the typical employment experiences of these parents under normal circumstances. Future research comparing prepandemic, mid-pandemic, and postpandemic data would be beneficial to differentiate between the pandemic’s specific effects and the longer-term employment trends for this population.

### Conclusions and Implications

This research examines employment and factors that affected fathers’ and mothers’ employment decisions. Our data show that a considerable proportion of mothers choose not to work, suggesting that women bear the majority of caring obligations. Gender inequality in employment exists in China and it is more pronounced than that in Western countries. The shortcomings of public assistance, the rigidity of the paid workforce regarding family medical leave, and the absence of compensation for informal care work make it challenging for women, particularly low socioeconomic status women, to find jobs. Policymakers should provide the necessary welfare supports, particularly for mothers caring for children with severe disabilities, allowing them to work fewer hours and offering a more flexible schedule or leave policy. Coordinating care for the entire family may result in improved health and economical outcomes. More maternal health care and social supports are required to increase the workforce involvement and well-being of these mothers. Furthermore, more research is needed to investigate the roles of fathers. A stronger gender equality ideology and more women-friendly policies are needed not only in China but around the world.

## Supplementary material

10.2196/59696 Multimedia Appendix 1 Gender inequalities in employment of parents caring for children with autism spectrum disorder in China.

## Abbreviations

ASD: autism spectrum disorder
HFA: high-functioning autism
LFA: low-functioning autism
OR: odds ratio
