# Supplementary material for: Gender Inequalities in Employment of Parents Caring for Children With Autism Spectrum Disorder in China: Cross-Sectional Study
Source: JMIR Pediatr Parent. 2024 Dec 17;7:e59696. doi: 10.2196/59696 (PMC11683506; doi:10.2196/59696)
Supplement: Multimedia Appendix 1 [file pediatrics-v7-e59696-s001.docx]

**Gender inequalities in employment of parents caring for children with autism spectrum disorders in China**

eTable 1. Distribution of sample by residence provinces

| Provinces | N | % |
| --- | --- | --- |
| Guangdong | 856 | 16.9 |
| Shandong | 341 | 6.7 |
| Anhui | 301 | 5.9 |
| Fujian | 293 | 5.8 |
| Jiangsu | 291 | 5.7 |
| Hunan | 274 | 5.4 |
| Henan | 272 | 5.4 |
| Hubei | 266 | 5.2 |
| Jiangxi | 254 | 5.0 |
| Hebei | 245 | 4.8 |
| Beijing | 187 | 3.7 |
| Jiangxi | 182 | 3.6 |
| Zhejiang | 178 | 3.5 |
| Sichuan | 143 | 2.8 |
| Shanxi | 127 | 2.5 |
| Shaanxi | 112 | 2.2 |
| Liaoning | 103 | 2.0 |
| Heilongjiang | 84 | 1.7 |
| Tianjin | 77 | 1.5 |
| Chongqing | 72 | 1.4 |
| Hainan | 68 | 1.3 |
| Shanghai | 64 | 1.3 |
| Guizhou | 58 | 1.1 |
| Jilin | 56 | 1.1 |
| Xinjiang | 50 | 1.0 |
| Inner Mongolia | 47 | 0.9 |
| Gansu | 29 | 0.6 |
| Yunnan | 24 | 0.5 |
| Ningxia | 12 | 0.2 |
| Xizang | 6 | 0.1 |
| Qinghai | 5 | 0.1 |
| Total | 5077 | 100.0 |

Abbreviation: N: number.

eTable 2. Data quality exploration

|  | **Study population** | | **The Second China National Sample Survey on Disability ^a^** | |
| --- | --- | --- | --- | --- |
|  | n | % | n | % |
| **District** |  |  |  |  |
| Eastern | 2,759 | 54.3 | 25,713^b^ | 62.3 |
| Central | 1,720 | 33.9 | 9,747^b^ | 23.6 |
| Western | 598 | 11.8 | 5,834^b^ | 14.1 |
| **Sex** |  |  |  |  |
| Boy | 4,244 | 83.6 | 32,130^b^ | 77.8 |
| Girl | 833 | 16.4 | 9,163^b^ | 22.2 |
| Sex-Ratio | 5.09 |  | 3.51^b^ |  |
| **total** | **5077** |  | **41,293^b^** |  |

Abbreviation: n: number.

a. The second national sample survey of the disabled began at 0:00 on April 1st, 2006 and ended on May 31st, 2006. The disabled sample was 252,6145. A total of **73** children aged 0-6 diagnosed with autism. All diagnoses were made by psychiatrists according to ICD-10.

b. weighted samples by Chinese population, which was not the actual number of screening people.

eTable 3. Variable definitions

| Workforce participation | Types | Definitions |
| --- | --- | --- |
| Work | Full-time | Being formally employed, working a minimum of 40 hours per week, and enjoying corresponding wages, social insurance, and welfare benefits |
|  | Flexible | Working hours are not fixed, usually less than 30 hours a week |
|  | Overtime | To work more than 40 hours per week |
|  | A long leave of absence | Be continuous absence exceeding 8 weeks, with salary |
| Non-work | Caregiving resignation | Quitted jobs due to children care (resignation after the child's diagnosis) Quitted jobs or did not work due to other reasons. |
|  | Others |  |

From : The National People's Congress Standing Committee of the People's Republic of China "Labor Law of the People's Republic of China". http://www.gov.cn/flfg/2005-08/29/content_28412.htm
